# Foxa2 and Pet1 Direct and Indirect Synergy Drive Serotonergic Neuronal Differentiation

**DOI:** 10.3389/fnins.2022.903881

**Published:** 2022-06-20

**Authors:** Begüm Aydin, Michael Sierk, Mireia Moreno-Estelles, Link Tejavibulya, Nikathan Kumar, Nuria Flames, Shaun Mahony, Esteban O. Mazzoni

**Affiliations:** ^1^Department of Biology, New York University, New York City, NY, United States; ^2^Interdisciplinary Sciences Department, Saint Vincent College, Latrobe, PA, United States; ^3^Developmental Neurobiology Unit, Instituto de Biomedicina de Valencia IIBV-CSIC, Valencia, Spain; ^4^Center for Eukaryotic Gene Regulation, Department of Biochemistry and Molecular Biology, The Pennsylvania State University, University Park, PA, United States

**Keywords:** neuronal differentiation, direct programming methods, Pet1, Foxa2, stem cell differentiation, transcription factor

## Abstract

Neuronal programming by forced expression of transcription factors (TFs) holds promise for clinical applications of regenerative medicine. However, the mechanisms by which TFs coordinate their activities on the genome and control distinct neuronal fates remain obscure. Using direct neuronal programming of embryonic stem cells, we dissected the contribution of a series of TFs to specific neuronal regulatory programs. We deconstructed the Ascl1-Lmx1b-Foxa2-Pet1 TF combination that has been shown to generate serotonergic neurons and found that stepwise addition of TFs to Ascl1 canalizes the neuronal fate into a diffuse monoaminergic fate. The addition of pioneer factor Foxa2 represses Phox2b to induce serotonergic fate, similar to *in vivo* regulatory networks. Foxa2 and Pet1 appear to act synergistically to upregulate serotonergic fate. Foxa2 and Pet1 co-bind to a small fraction of genomic regions but mostly bind to different regulatory sites. In contrast to the combinatorial binding activities of other programming TFs, Pet1 does not strictly follow the Foxa2 pioneer. These findings highlight the challenges in formulating generalizable rules for describing the behavior of TF combinations that program distinct neuronal subtypes.

## Introduction

The complex functions of the nervous system require an exquisite repertoire of specialized neuron types primarily defined by their transcriptome. Effector genes contributing to neuronal terminal features are the components of the transcriptome that define the functionality of the neuron, from functions common to all neurons (cell polarity, excitability, etc.) to those specific for neuronal types (neurotransmitter receptors, transporters, biosynthetic enzymes, etc.). With the growing collection of induced pluripotent and embryonic stem cells carrying neurodegenerative genotypes – for example the iPSC Neurodegeneration Initiative (iNDI) project – there is a need to establish rules that govern transcription factor-induced neuronal programming to differentiate them into diverse neuronal types with high accuracy and efficiency (Wapinski et al., 2013).

Transcription factors (TFs) are the main players controlling transcriptional activity during cell-type specification. In recent years, reprogramming, direct programming, and transdifferentiation experiments have taken advantage of this principle to impose cell type-specific gene regulatory programs (Morris, 2016; Aydin and Mazzoni, 2019). TF-induced direct programming into neurons has gained popularity due to its efficiency and scalability. Direct neural programming can be rationalized as a two-module process, consisting of inducing a “generic” neuronal fate (axonal growth, synaptic machinery, etc.) and specifying neuronal type-specific gene expression controlling features such as neurotransmitter biosynthesis. The expression of the pro-neuronal TFs Ascl1 and Neurog2 induce neuronal fate from pluripotent stem cells (Busskamp et al., 2014; Aydin et al., 2019). Although Ascl1 and Neurog2 induce their own neuronal subtype bias, combining the pro-neuronal TF with other neuronal fate-specific TF combinations refines the transcriptome and accelerates terminal neuron-type specific fate conversion (Aydin et al., 2019; Lin et al., 2021). For example, pairing Neurog2 with Isl1 and Lhx3 drives spinal motor neuron fate from pluripotent stem cells (Hester et al., 2011; Mazzoni et al., 2013). On the other hand, combining Ascl1 with Lmx1a and Nurr1 induces midbrain dopaminergic fate (Caiazzo et al., 2011).

Because it provides a well-controlled cellular environment amenable for precise time series and experimental perturbations, direct programming has become a favored strategy to investigate how TFs control cell fate. The proneural Ascl1 or Neurog2 behave as pioneer TFs (Castro et al., 2006; Wapinski et al., 2013; Soufi et al., 2015; Smith et al., 2016; Aydin et al., 2019). Thus, they can access sites on the genome even when they are occluded by nucleosomes and are therefore able to induce neuronal fate from both pluripotent and terminally differentiated cells (Farah et al., 2000; Parras et al., 2002; Castro et al., 2006). The binding of other neuronally expressed TFs can be affected by the accessibility landscape established by Ascl1 or Neurog2. For example, the broadly expressed Ebf2 and Brn2 bias their binding targets toward regions made accessible by pro-neuronal TFs (Castro et al., 2006; Wapinski et al., 2013; Aydin et al., 2019). However, neuron type-selecting TFs do not always bind to regions bound by proneural TFs. The Isl1 and Lhx3 TF pair dimerize during motor neuron direct programming and do not follow the Neurog2-established TF accessibility (Velasco et al., 2017). In turn, in a feed-forward transcriptional logic, Isl1-Lhx3 binding changes as differentiation progresses following the changing accessibility created by the Onecut TFs (which also have pioneer activity) induced by Neurog2 (Rhee et al., 2016; Velasco et al., 2017; van der Raadt et al., 2019). Expression of non-pioneer TFs can also modify the binding landscape of a given TF and its direct targets. For example, swapping Lhx3 with Phox2a allows Isl1 to target a new set of regulatory elements and program a different motor neuron type (Mazzoni et al., 2013). Thus, Isl1-Lhx3 and Isl1-Phox2a target enhancers to induce neuronal type-specific gene expression in two related neuronal types. These examples show the wide range of strategies used to implement specific neuron fates and the importance of both direct and indirect interactions between TFs. Thus, much work remains to be done to elucidate which rules apply to various TF combinations, including possible conflicts when coexpressing multiple pioneer TFs.

Monoamine neurotransmitters contain one amino group connected to an aromatic ring by a two-carbon chain. In vertebrates, they include mainly catecholamines (dopamine, noradrenaline, adrenaline) and serotonin. Each monoaminergic neuron type is classified by coordinated expression of a set of genes that control the synthesis and transport of specific monoamines, and some of these genes are shared among all monoaminergic neurons (Flames and Hobert, 2011). However, how these sets of genes are regulated during monoaminergic neuron differentiation is unclear. Ascl1 is prominently expressed in the monoaminergic central and peripheral neural progenitors, and it is both necessary and sufficient to promote neurogenesis (Pattyn et al., 2004; Vasconcelos and Castro, 2014). Another pioneer TF, the Forkhead family TF Foxa2 is expressed in midbrain dopaminergic neurons and ventral hindbrain serotonergic progenitor domains (Vasconcelos and Castro, 2014). Reciprocal repression between homeodomain protein Phox2b and Foxa2 mediates the progenitor switches from visceral motor neuron fate into serotonergic fate (Pattyn et al., 2000). In this region, prolonged Foxa2 expression in progenitors is required for the activation of serotonergic TFs such as Gata2, Lmx1b, and Pet1 (also known as Fev) (Jacob et al., 2007). The LIM homeodomain TF Lmx1b is expressed along the ventral midbrain and hindbrain, and it is also important for the development of both dopaminergic and serotonergic neurons. In Lmx1b homozygous mutants, serotonergic neuron precursors fail to activate the expression of Tph2/tryptophan hydroxylase, Sert/serotonin reuptaker, and Vmat2/vesicular monoamine transporter and fail in the synthesis of serotonin (5-HT) even though the number of serotonergic precursors does not change (Ding et al., 2003). Moreover, Lmx1b is also required for correct midbrain dopaminergic neuron specification (Smidt et al., 2000; Lin et al., 2009; Yan et al., 2011). Finally, Pet1 is an ETS transcription factor expressed in central nervous system postmitotic serotonergic neurons and is required for normal serotonergic neuron differentiation, function, and fate maintenance (Hendricks et al., 2003; Maurer et al., 2004). Thus Ascl1, Foxa2, and Lmx1b are required for both dopaminergic and serotonergic specification, while Pet1 is exclusively involved in serotonergic induction. *In vivo*, this set of TFs acts at different stages in the differentiation process. Ascl1 and Foxa2 are pioneer factors acting mainly in progenitors, while Lmx1b and Pet1 act in postmitotic cells to directly induce neuron-type specific features (Hendricks et al., 1999; Cheng et al., 2003; Pattyn et al., 2004; Jacob et al., 2007). In addition, expression of both Lmx1b and Pet1 is sustained throughout the life of the animal and is required to maintain neuron fate (Liu et al., 2010; Donovan et al., 2019). Considering their postmitotic, direct and terminal actions, Lmx1b and Pet1 can be classified as terminal selectors for serotonergic fate.

We deconstructed a monoaminergic TF combination to interrogate how adding TFs shapes their activity and neuronal programming. The Ascl1 + Lmx1b + Foxa2 + Pet1 (ALFP) TF combination transdifferentiates human fibroblasts toward serotonergic neuron fate (Xu et al., 2016). This study focuses on a simple system programming neuronal fate from mouse pluripotent stem cells by increasing the TF number from induced (i) Ascl1 only (iA) to iALFP. As expected, all combinations generated neurons efficiently due to the inclusion of the proneural Ascl1. Based on typical dopaminergic and serotonergic marker immunocytochemistry, iALFP induces serotoninergic fate at higher percentages than do differentiating cells expressing iA, iAL, iALP, or iALF. The fact that iALFP expression differs from a simple superposition of iALF and iALP suggests Pet1 and Foxa2 act synergistically. Thus, we investigated how the induction of different TF combinations affects neuronal gene expression, TF binding, and chromatin accessibility. We find that each TF combination shows a specific gene expression profile. iALFP is the most different from naive embryoid bodies (EB) and the best inducer of serotonergic effector gene expression. As expected for a pioneer TF, Foxa2 does not change its binding location when expressed with Pet1. On the other hand, Pet1 binds to different sites in the presence of Foxa2. Although the few Foxa2-Pet1 co-bound sites seem to be biologically relevant, Foxa2 and Pet1 bind mostly independently to different genomic locations.

## Results

### Foxa2 and Pet1 Act in Concert With Ascl1 and Lmx1b to Induce Serotonergic Identity

**FIGURE 1:**
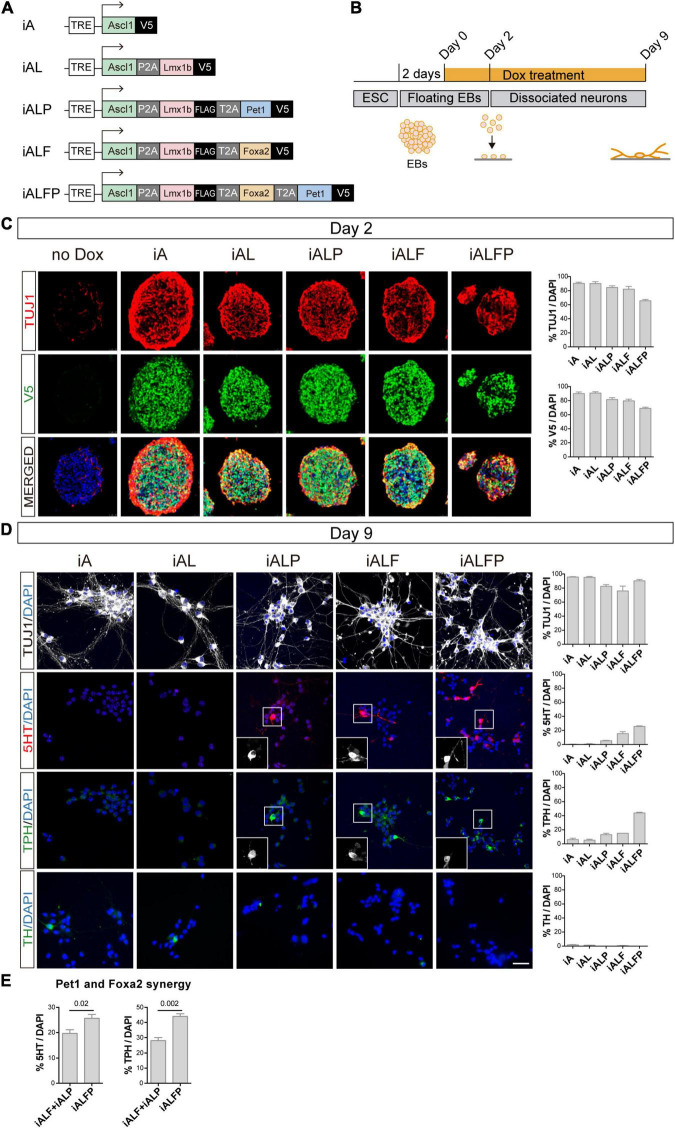
Dissection of the combinatorial action of serotonergic TFs. **(A)** TF combinations induced in ESC. **(B)** TF induction, differentiation, and analysis outline. Doxycycline treatment is started 2 days after floating EB preparation. EBs are dissociated and plated 2 days later (Day 2) and cultured in the presence of doxycycline for 7 more days (Day 9 analysis). **(C)** Micrographs and quantification of TF induction (monitored by V5 expression) and neuronal fate (monitored by beta tubulin 3,TUJ1 staining) in EBs 2 days after doxycycline treatment. Broad iTF and neuronal differentiation induction in all cell lines. **(D)** Micrographs and quantification of neuronal (TUJ1), serotonergic (5HT and TPH) and catecholaminergic (TH) fate after 7 days of neuronal differentiation. Catecholaminergic expression is absent in all lines, while serotonergic markers are highest in iALFP. **(E)** Measurement of synergistic effects in iAFLP line. The addition of iALF + iALP serotonergic or TPH expression is significantly lower than values found in iALFP, suggesting synergistic effects between Pet1 and Foxa2. Scale bar: 50 μm.

To study how TF combinations induce neuronal and serotoninergic differentiation, we constructed a series of mouse isogenic inducible embryonic stem cell lines (iESCs), inserting each TF combination at the HPRT locus (Iacovino et al., 2011; Mazzoni et al., 2011). Self-cleaving 2A peptides between coding sequences allowed for simultaneous and equimolar induction of TFs in each inducible cell line (Mazzoni et al., 2013). In total we built the following inducible lines: Ascl1 (iA), Ascl1 + Lmx1b (iAL), Ascl1 + Lmx1b + Foxa2 (iALF), Ascl1 + Lmx1b + Pet1 (iALP), and Ascl1 + Lmx1b + Foxa2 + Pet1 (iALFP) (Figure 1A). The last TF in each combination was tagged with V5. iESCs were detached and allowed to form EB and 2 days later, TFs were induced by adding 3ug/ml of Doxycycline (Dox) to initiate differentiation (Figure 1B). All cell lines induced TF expression at high percentages after 2 days of Dox and efficient cleavage of the multicistronic constructs (Figure 1C, Supplementary Figure 1A, and Supplementary Table 1). As evidenced by efficient neuronal differentiation (TUJ1, Figure 1C and Supplementary Table 1), adding multiple TFs in a polycistronic construct did not inhibit Ascl1 pro-neuronal activity. We note that as the inducible construct became larger and more complex, there was a slight decrease in TF induction (Figure 1C and Supplementary Table 1). However, all combinations were very effective at inducing neuronal fate, with more than 95% of the cells expressing the construct becoming neurons in all lines (Supplementary Table 1).

Two days after Dox treatment, we dissociated the EBs into single-cell suspension and plated them as a monolayer to measure neuronal conversion and induction of monoaminergic fate (Figure 1B). TUJ1 staining revealed once more that each iESC line differentiates to a neuronal fate efficiently and maintains neuronal fate after 7 days in culture (Figure 1D and Supplementary Table 1). We then stained these neurons with antibodies against serotonin (5HT), Tryptophan hydroxylase (TPH), and tyrosine hydroxylase (TH) to quantify serotonergic (5HT and TPH) and catecholaminergic fate (TH is expressed in dopaminergic, adrenergic and noradrenergic neurons) (Figure 1D and Supplementary Table 1). None of the TF combinations induced TH in a sizable fraction of the cells. However, there was an increase in markers for serotoninergic fate as the TF combination became more complex, from iA to iALFP (Figure 1D and Supplementary Table 1). Neither Ascl1 alone (iA), nor in combination with Lmx1b (iAL), induced 5HT or TPH. The addition of Pet1 or Foxa2 to iAL (iALP and iALF, respectively) was sufficient to induce serotonergic staining and TPH expression (Figure 1D and Supplementary Table 1). Interestingly, the full TF set (iALFP) induced serotoninergic markers at higher levels than the simple addition of iALP + iALF effects (Figure 1E and Supplementary Table 1). Thus, we conclude that iALFP induces neurons expressing serotonergic fate when differentiating ESCs. Moreover, Pet1 and Foxa2 are required and seem to act synergistically to induce this specific neuron-type fate.

### Foxa2 and Pet1 Make Both Independent and Synergistic Contributions to Gene Expression

**FIGURE 2:**
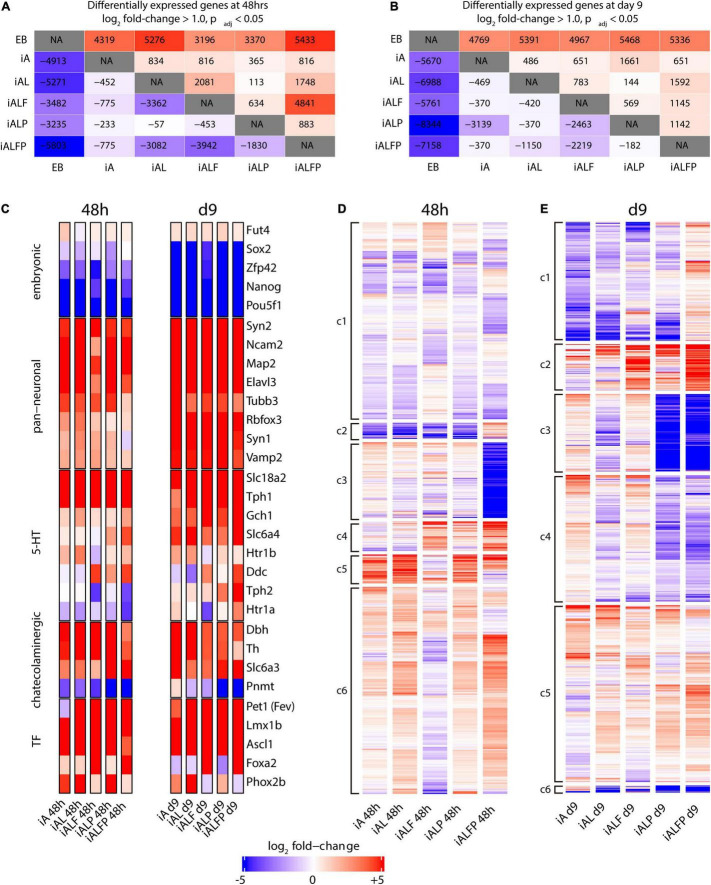
Transcription profile of induced neurons at differentiation days 2 and 9. A, B. Counts of up- and downregulated genes under different exogenous transcription factor constructs at 48 h **(A)** and 9 days **(B)** post-induction. Conditions were compared in a pairwise fashion using DESeq2. The upper half of the diagonal is upregulated genes, the lower half is downregulated genes. **(C)** Heatmap of diagnostic gene expression at 48 h and 9 days post TF induction. Slc18a2 a.k.a. Vmat2; Slc6a4 a.k.a. Sert; Slc6a3 a.k.a. Dat. **(D,E)** Bulk RNA-seq heatmaps under different exogenous transcription factor constructs at 48 h **(D)** and 9 days **(E)** post-induction. At each timepoint, genes that were either upregulated or downregulated in all five conditions were removed, and genes that did not have a log fold change of at least 1.0 with an adjusted *p*-Value of 0.05 according to DESeq2 in at least one of the five transcription factor conditions were also removed, leaving 6393 genes at 48 h and 3970 genes at day 9. These remaining genes were clustered using K-means clustering using the R kmeans function. Transcripts were assigned to six clusters (c1 to c6) based on the expression pattern across all conditions.

To characterize the contributions that Foxa2 and Pet1 make to the serotonergic expression program, we performed bulk RNA-seq experiments in EBs and in each of the five cell lines after inducing expression of the various TF combinations 2 and 9 days after Dox treatment to measure the initial transcriptional response and the terminal neuronal fate. Figures 2A,B show the numbers of up- and down-regulated genes (log_2_ fold change ≥ 1.0, adjusted *p*-value < 0.05) for all pairwise comparisons at 48 h and 9 days post-induction, respectively.

As expected, all TF inductions produce substantial numbers of differentially expressed genes compared with EBs, with the full TF set (iALFP) inducing the largest transcriptional difference vs. EBs (Figures 2A,B). However, each TF combination generates unique patterns of gene expression. The iAL line displays relatively little change in expression compared with iA (834 genes upregulated, 452 down-regulated at 48 h), suggesting that Lmx1b does not substantially modulate the broad proneural expression program initiated by Ascl1. However, we noticed high levels of endogenous Lmx1b expression in the iA line (Figure 2C), which might partly explain their transcriptional similarities. The addition of Pet1 (iALP) causes modest increases in the number of genes differentially expressed at either day 2 (113 up, 57 down from iAL) or day 9 (144 up, 370 down from iAL) (Figures 2A,B). The expression impact of the exogenous Pet1 may also be reduced since the iAL line induced some levels of endogenous Pet1 (Figure 2C).

Endogenous Foxa2 expression levels are low in iA, iAL, and iALP. The addition of exogenous Foxa2 had a substantial impact on gene expression. The iALF line has a relatively large number of expression differences compared with iAL (2,081 up, 3,362 down at 2 days, Figure 2A). Surprisingly, while Pet1 does not significantly affect expression when expressed alongside Ascl1 and Lmx1b, it strongly modulates the gene expression program induced by iALF. The induction of all four TFs together (iALFP) produces an expression pattern that is different from iALF at both day 2 (4,841 up, 3,942 down) and day 9 of differentiation (1,145 up, 2,219 down) (Figures 2A,B). These results resonate with the hypothesis that Pet1 and Foxa2 act synergistically. We also noticed that although cell-line specific gene expression profiles are found both at day 2 and day 9 of differentiation (Figures 2A,B), differences are exacerbated at earlier time points suggesting convergence toward more similar neuron fates.

Next, we focused on the expression of diagnostic genes for pluripotency, pan-neuronal or monoaminergic cell fate. As expected, pluripotency genes were downregulated upon TF induction (Figure 2C). Concomitantly pan-neuronal gene expression was activated in all cell lines at 2 days and at higher levels and broadly at 9 days (Figure 2C). In addition, catecholaminergic effector gene expression [tyrosine hydroxylase (Th), dopamine transporter (Slc6a3) and dopamine beta hydroxylase (Dbh)] is observed in all cell lines at both differentiation times. At two days, core genes coding for 5HT biosynthesis was higher but incomplete in the iALFP line. However, this marker set increased in iALFP by 9 days of differentiation (Figure 2C). In mammals, the Tph1 and Tph2 genes code for the tryptophan hydroxylase, regulating the rate-limiting step for 5HT biosynthesis. *In vivo*, Tph2 but not Tph1 is expressed in hindbrain serotonergic neurons. We find high Tph1 expression in all cell lines at both differentiation time points, however, Tph2 expression is only induced by iALFP at 9 days of differentiation (Figure 2C). Thus, the serotonergic signature settles in iALFP as neurons mature in culture.

We noted that Th is slightly repressed in iALFP at longer differentiation times. The presence of Th transcript contrasts with the lack of TH staining (Figure 1D) and might indicate additional layers of posttranscriptional control, as has been described *in vivo* (Xu et al., 2007). Expression of noradrenergic specific enzyme Phenylethanolamine-N-methyltransferase (Pnmt) is slightly induced in iA line but highly repressed in iALP and iALFP at 9 days of differentiation (Figure 2C). Foxa2 is critical for serotoninergic development in the hindbrain by suppressing Phox2b TFs (Jacob et al., 2007). Recapitulating this regulation, iALF and iALFP cells do not express Phox2b induced by iA, iAL, and iALP. Foxa2 repression of Phox2b is seen at 2 and 9 days of doxycycline treatment but is stronger at later time points (Figure 2C). In summary, all cell lines equally repress pluripotency and induce generic neuronal gene expression. Although alternative monoaminergic fates are not entirely silenced, the ALFP TF combination is the one that more closely reproduces serotonergic effector gene expression, particularly at longer differentiation times.

To further explore the differences in expression programs more broadly, we performed K-means clustering on all genes with a log_2_ fold change of at least ± 1.0 in at least one of the 5 cell lines compared to EB (Supplementary Figure 1). The five cell lines have broadly similar transcription regulation patterns from EBs, consistent with the notion that neuronal differentiation drives most transcriptional changes. To separate the neuronal component from a possible neuronal subtype signature, we removed all either upregulated or downregulated genes in all five cell lines and re-clustered the remaining genes. The resulting heatmaps at day 2 (Figure 2D) and day 9 (Figure 2E) illustrate the unique impacts on expression caused by each TF combination. A list of GO terms for each cluster can be found in Supplementary Tables 3, 4.

We first focused on the analysis of day 2 as it better reflects the direct actions of TF combinations. The expression clusters found at day 2 include several expression patterns that are present in the iALFP line, but not in either the iALF or the iALP lines (Supplementary Tables 2–4 for clusters’ GO terms at day 2 and day 9 respectively). For example, cluster 2 shows a group of 186 genes that are generally downregulated in all cell lines except for iALFP. Cluster 3, in contrast, contains genes that are strongly downregulated only in the context of iALFP and contains genes associated with GABA transporter activity according to Enrichr (Xie et al., 2021), many pseudogenes, and several Hox genes expressed in the most posterior rhombomeres (Hoxb2, Hoxb5, and Hoxa3). These two gene clusters suggest that Pet1 and Foxa2 synergistically create a unique expression program when expressed alongside Lmx1b and Ascl1. Other expression clusters suggest somewhat independent roles for Foxa2 and Pet1 in activating subsets of genes. Cluster 5 contains genes whose expression is inverted by the addition of Pet1, that is genes upregulated in iALF that are downregulated in iALFP and vice versa genes downregulated in iALF that are upregulated in iALFP. This cluster is enriched for genes associated with neuronal differentiation. Several of them, including Cnr1, Cyfip2, Fgf13, Col25a1, and Slc17a8, are downregulated in serotonergic neurons in the Lmx1b mutant mice (of note, Lmx1b is also upstream of Pet1 expression) (Donovan et al., 2019). Cluster 4 contains upregulated genes in both iALF and iALFP, suggesting that they are downstream of Foxa2. This cluster is enriched for genes associated with dopaminergic and serotonergic neurogenesis (Ddc, Shh, Lmx1a, En1, Gli1, Nkx2.2) and genes associated with axon guidance in serotoninergic neurons (Donovan et al., 2019). Many Cluster 6 genes are downregulated in iALF, but upregulated in iALP and iALFP, suggesting that Pet1 overexpression overrides an apparent repressive effect of Foxa2 to activate these genes.

The expression clusters found at day 9 also reflect differences in each cell line, including patterns present in the iALFP line, but not in either the iALF or the iALP lines (Figure 2E). However, enriched GO terms did not reach statistical significance. We found that many of the genes from Cluster 4 at 48h (those upregulated in both iALF and iALFP) are also present in differential expression clusters at day 9, particularly in clusters 1 and 2 corresponding to genes with higher expression in ALFP than in ALF or ALP. This gene set is enriched for cadherin-mediated cell adhesion. Ddc, the effector gene required for serotonin biosynthesis, is also present in this group of genes along with additional genes expressed in mouse brain serotonergic neurons (Zeisel et al., 2018), such as Renbp, Naip6, Macc1, Iqcf5, II1r1, Hsd367, Foxa1, Cthrc1, Crybg3, Col7a1 and Clps. Finally, FPKM values for Th, Tph1 and Tph2 expression confirms synergistic actions of Foxa2 and Pet1 in Th repression and Tph activation (Supplementary Figure 1).

In total, transcriptomic analysis suggests that adding serotonergic TFs to Ascl1 induced gene expression patterns associated with serotonergic fate. We note that Pet1 and Foxa2 are required to independently and synergistically control different gene expression modules.

### Single-Cell RNA-Seq Confirms Mixed Monoaminergic Fate Induction at Early Differentiation Time Points

**FIGURE 3:**
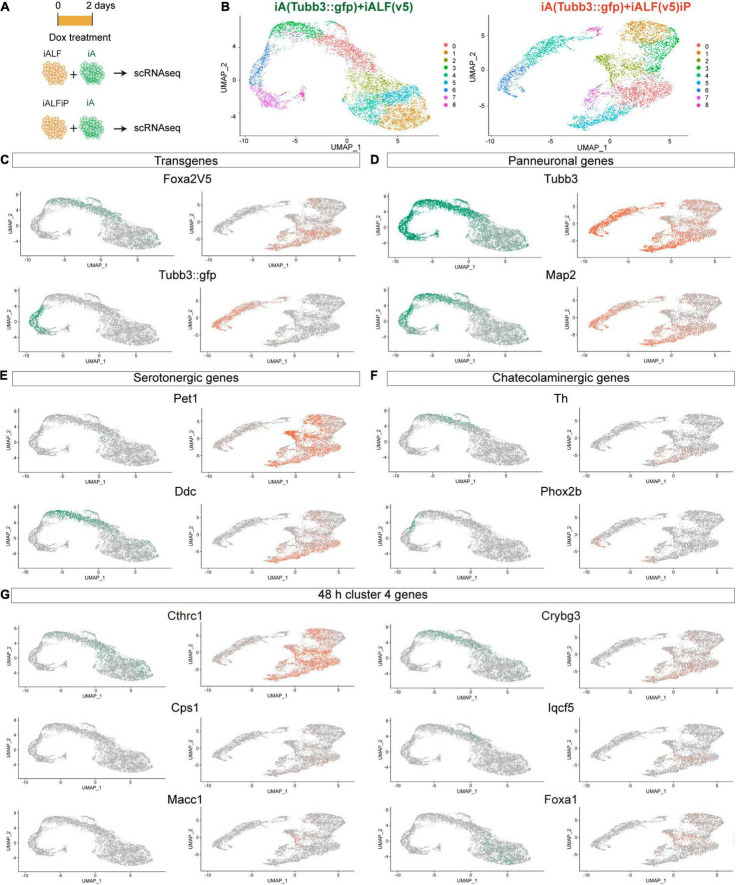
Single-cell RNA-seq of iALF and iALFiP neurons at differentiation day 2. **(A)** Experimental outline. iALF and iALFiP cells were mixed with Tubb3:GFP-expressing iA cells for comparison. **(B)** UMAP visualization of iALF and iALFiP clustering. Clusters are shown in colors and numbered from 1 to 8. **(C–G)** Projection of specific genes in panel **(B)**. **(C)** Transgene expression clearly separates iA (Tubb3:GFP+) from iALF and iALFiP. **(D)** The transgene expression levels corelate with neural differentiation states suggesting a differentiation cline across cells. **(E,F)** Both lines contain cells expressing serotoninergic genes but iALFiP represses Phox2b. **(G)** Projection of representative Cluster 4 (Figure 2D) on the single cell clusters. **(C–G)** Green = expression in iALF + Tubb3:GFP, Orange = iALFP + Tubb3:GFP.

The bulk RNA-seq results show that TF induction generated a mixture of different monoaminergic fates. To dissect if heterogeneity of bulk gene expression corresponds to different cell populations or to mixed neuron-type fate induction in single cells and to try to deconvolve the effects of Pet1-Foxa2 synergy, we performed single-cell RNA-seq experiments (scRNA-seq) 2 days after Dox induction in iALF and iALFP. To avoid possible artifacts induced by inefficient 2A peptide cleavage producing unprocessed Foxa2-Pet1 TF proteins, we created a new line where Pet1 is driven by an independent Dox-inducible promoter (iALFiP). Neuronal and serotonergic staining at 9 days of doxycycline treatment is similar to iALFP (Supplementary Table 1). To measure the difference with neurons induced by Ascl1 only, we spiked iALF and iALFiP single-cell suspensions with a fluorescently labeled iA line immediately before scRNA-seq encapsulation (Figures 3A,B). Confirming the strong effect of adding TFs to Ascl1, the iALF and iALFiP cells labeled by the Foxa2-V5 transgene clustered away from iA cells labeled by Tubb3:GFP in a dimensional reduction representation (Figure 3C).

iALF and iALFiP combinations contained cells in different states of neuronal differentiation, as seen by a range of endogenous Tubb3 and Map2 transcript levels (Figure 3D). Expression of most serotonin and catecholamine biosynthesis pathway genes are not or almost not detectable at this early stage of differentiation, including serotonin exclusive Tph2 and Slc6a4 (a.k.a. Sert) genes, catecholaminergic exclusive Slc6a3 (a.k.a Dat), and Dbh or shared Gch, and Slc18a2 (a.k.a. Vmat2). However, scRNA-seq reveals expression for Ddc (commonly expressed by serotonergic and catecholaminergic genes) and Th (not expressed by serotonergic neurons) (Figures 3E,F). Ddc expression is present in iALF and iALFiP cells with high Tubb3 and Map2 expression levels but absent from iA cells. Th expression also coincides with high levels of Tubb3 and Map2, although its expression seems lower and in fewer cells than Ddc expression, particularly in iALFiP.

As expected from bulk RNA-seq, iALF and iALFiP cells repress Phox2b expression (Figure 3F). Next, we analyzed scRNA-seq expression for genes classified in cluster 4 in our bulk RNA-seq experiments. This gene set contains upregulated genes in both iALF and iALFiP, suggesting that they are downstream of Foxa2 and are enriched for dopaminergic and serotonergic neurogenesis genes. We selected some genes with detectable expression in serotonergic neurons *in vivo* (Zeisel et al., 2018). These genes are expressed in iALF and iALFiP but not induced in iA. Some of them show higher or broader expression in iALFiP compared to iALF (such as Cthrc1, Cps1 and Macc1) (Figure 3G), while others (such as Crybg3, Iqcf5 or Foxa1) seem more similarly expressed in iALF and iALFP (Figure 3G).

In total, the scRNA-seq experiments showed that both TF combinations induce a collection of cells with varying states of maturation 48 h after Dox induction. As expected, neurons further along the differentiation pathway express genes associated with terminal neurotransmitter fate (Th and Ddc) supporting maturation as a key factor to induce the terminal serotoninergic markers. Thus, most of the effector genes are still undetectable at this early differentiation stage. Broad Th expression suggest mixed monoaminergic fate induction at early time points. Although iALF and iALFiP cells induce similar neuronal fates overall, iALFiP generates a higher percentage of cells with genes associated with serotonergic fate, such as Cthrc1, Cps1 and Macc1.

### Foxa2 and Pet1 Bind Mostly Independently to the Genome

**FIGURE 4:**
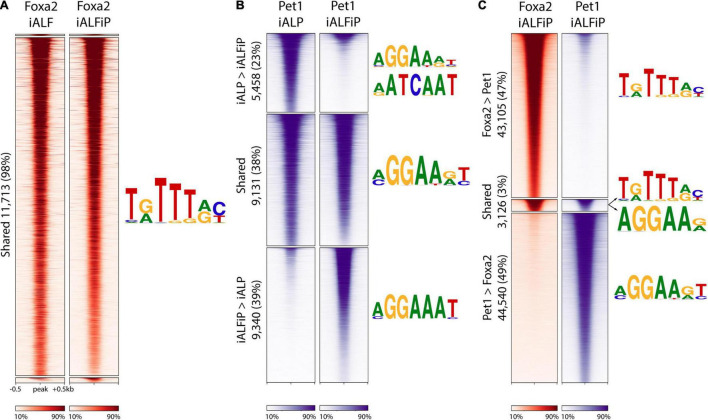
The presence of Foxa2 affects the location of Pet1 binding. **(A)** Heatmap of ChIP-seq reads at Foxa2 binding sites in ALF and ALFiP induction backgrounds after 12 h. A 1 kb window around the peak center is plotted. The plots are divided top-to-bottom based on the preferential binding patterns ALF > ALFiP, shared, and ALFiP > ALF, according to MultiGPS. The sequence logos of the motifs detected by MEME-ChIP are shown to the right of the region. **(B)** Heatmap of ChIP-seq reads of Pet1 binding sites in ALP and ALFP background. **(C)** Heatmap of ChIP-seq reads at Foxa2 (red) and Pet1 (blue) binding sites in ALFP background. The color scaling in all heatmaps vary between the read counts observed in the 10th and 90th percentiles of bin.

Since Pet1 and Foxa2 appear to synergistically regulate some sets of genes after only 2 days of differentiation (Figure 2D), we asked whether they interact with each other at their DNA-binding targets. We thus performed ChIP-seq on Foxa2 in the iALF and iALFiP cell lines and Pet1 in the iALP and iALFiP cell lines, where all experiments were performed after 2 days of TF combination induction. Although Foxa2 tends not to bind proximal to transcription start sites, Pet1 has a more evenly distributed binding (Supplementary Figure 2). All sets of binding sites are enriched for appropriate cognate DNA-binding motifs. MEME-ChIP motif discovery analysis finds Foxa2’s cognate binding motif enriched at Foxa2’s binding sites and the expected ETS family motif enriched in all three Pet1 binding site categories (Figure 4).

We first asked if the differences between iALF and iALFP (and iALFiP) transcriptional output are explained by Pet1 modifying Foxa2’s genomic binding. Foxa2 binding locations appear to be unaffected by the presence of Pet1, as the vast majority of Foxa2 sites display similar levels of ChIP enrichment in the iALF and iALFiP lines (Figure 4A). In contrast, over 60% of Pet1 sites display significant differential enrichment between the iALP and iALFiP conditions (23% are preferred in iALP while 39% are preferred in iALFiP and 38% are shared) (Figure 4B). While this suggests that Pet1’s binding targets are modified by Foxa2 expression, only a fraction of Pet1’s differential binding locations are directly attributable to a shift toward Foxa2’s binding sites. Specifically, of the 9,340 sites preferentially bound by Pet1 in iALFiP vs. iALP, only 1891 (20%) overlap Foxa2 binding locations. Thus, at most, only 20% of Pet1 differential binding could be directly affected by Foxa2 binding in cis. And considering all Pet1 and Foxa2 binding sites in ALFP only 3% are shared between the two TFs (Figure 4C).

To find sequence features that may explain the shift in Pet1 binding sites across cell lines, we divided all Pet1 bound sites into iALP > iALFiP, iALP = iALFiP and iALP < iALFiP and turned to the SeqUnwinder discriminative motif-finding platform (Kakumanu et al., 2017). SeqUnwinder identifies two Forkhead-like motifs that distinguish the iALFiP-preferred Pet1 sites from the other categories (Supplementary Figure 2). This is consistent with the 20% overlap of those Pet1 binding sites with Foxa2 binding, as noted above. In contrast, the iALP-preferred Pet1 sites contain discriminative motifs that match Homeodomain TFs, including a motif preferred by Onecut TFs (Supplementary Figure 2). Of note, when we compared Pet1 binding sites with our previously characterized Onecut2 binding sites (measured in iA cells after 48 h of induction, Aydin et al., 2019), we found a substantially higher overlap with iALP-preferred Pet1 sites (27%) compared with iALFiP-preferred sites (<1%). We further measured the binding of Lmx1b in iAL cells, finding 5,151 binding sites in total (Supplementary Figure 3), and again found a higher overlap with iALP-preferred Pet1 sites (12%) compared with iALFP-preferred sites (<1%).

Consistent with it being a pioneer TF, the ChIP-seq analyses support a model in which Foxa2 binds directly to cognate sites and is largely unaffected by the over-expression of Pet1. On the other hand, Foxa2 heavily perturbs Pet1’s binding targets. Surprisingly, only a small fraction of Pet1 binding changes could be explained by Foxa2 pioneer activity in cis. This fraction of shifted binding sites could be explained by Pet1 moving away from binding alongside other pioneer TFs expressed in neurons, like Onecut, toward binding alongside Foxa2. Nevertheless, most Pet1 sites preferentially bound in iALFiP are occupied independently of Foxa2 binding, likely interacting with additional unidentified TFs downstream of Foxa2.

### Both Foxa2 and Pet1 Bind to Relatively Inaccessible Regions on the Genome

**FIGURE 5:**
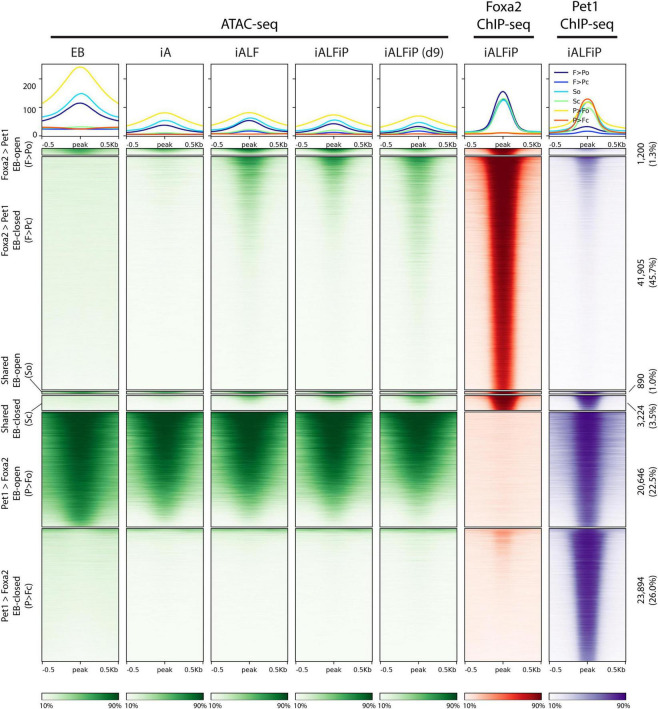
Comparison of ATAC-seq profiles with iALFiP Foxa2 and Pet1 binding categories. ATAC-seq heatmaps plot EB, iA (48 h), iALF (48 h), iALFiP (48 h), and iALFiP (d9) ATAC-seq signals over the Foxa2 and Pet1 binding categories displayed in Figure 4C. Each Foxa2 and Pet1 binding category is first split into two groups according to overlap with ATAC-seq domains in the preexisting EB cell state. Thus, six categories of sites are plotted. Panels above heatmaps display average ATAC-seq or ChIP-seq signals in each of the six categories. The color scaling in all heatmaps vary between the read counts observed in the 10th and 90th percentiles of bin.

The previously described Fox TF pioneer activity motivated us to ask if Foxa2 behaves similarly in this context and if Pet1 acts as a pioneer or not. To that end, we performed ATAC-seq experiments in the EB, iA (48 h), iALF (48 h), iALFiP (48 h), and iALFiP (day 9) conditions. A large majority (89%) of Foxa2 binding sites are inaccessible in the preexisting EB cells (Figure 5). Consistent with Foxa2’s known pioneering activity, Foxa2 binding increases chromatin accessibility at many sites in both iALF and iALFiP cell lines, and this accessibility is maintained and strengthened in day 9 iALFiP neurons (Figure 5).

**FIGURE 6:**
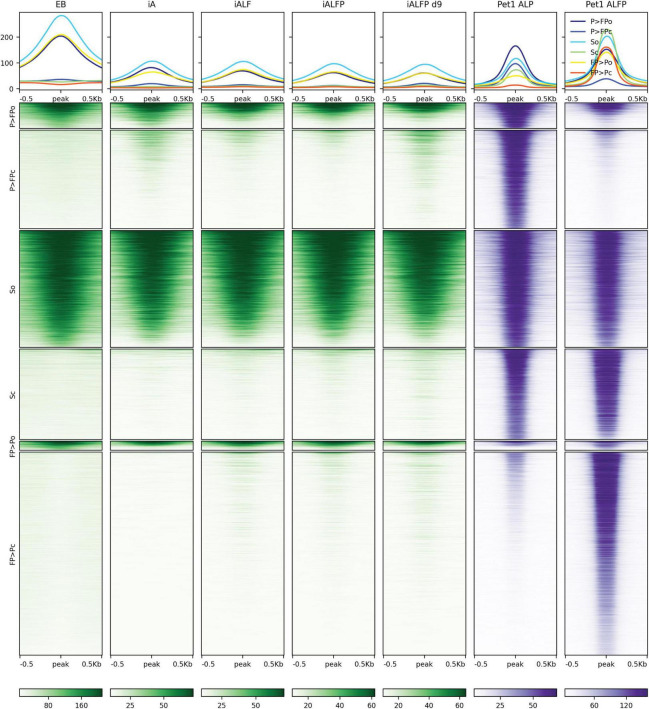
Comparison of ATAC-seq profiles with iALFiP Foxa2 and Pet1 binding categories. ATAC-seq heatmaps plot EB, iA (48 h), iALF (48 h), iALFiP (48 h), and iALFiP (d9) ATAC-seq signals over the iALP and iALFiP Pet1 binding categories displayed in Figure 4B. Each Pet1 binding category is first split into two groups according to overlap with ATAC-seq domains in the preexisting EB cell state. Thus, six categories of sites are plotted. Panels above heatmaps display average ATAC-seq or ChIP-seq signals in each of the six categories. The color scaling in all heatmaps vary between the read counts observed in the 10th and 90th percentiles of bin.

Pet1 displays a more complex association with accessibility. Over half of Pet1 binding sites in iALFiP cells are devoid of accessibility signatures in the preexisting EB cells, suggesting that Pet1 can bind to inaccessible chromatin (Figure 6). Intriguingly, and in contrast to the stereotypical behavior of a pioneer TF, Pet1 binding sites do not gain accessibility following Pet1 binding. Of the Pet1 binding sites that have preexisting accessibility in EB cells, most are bound by Pet1 in both iALP and iALFiP cell lines and thus fall under the “shared” or “iALP = iALFiP” category of Pet1 binding (Figure 6). Most iALP-preferred Pet1 sites are inaccessible in EB, and while some of these sites display increased accessibility in iA (48 h) and iALFiP (9 days) cells, most do not in iALF and iALFiP at 48h.

In summary, Foxa2 mainly binds to inaccessible chromatin regions and increases accessibility. We cannot rule out that Pet1 plays a pioneering role at a subset of its binding sites, but it is unlikely given the overall trend that the ATAC-seq signal does not increase at Pet1 bound sites.

### Foxa2 and Pet1 Binding Sites Are Associated With Neuronal Subtype Specification

**FIGURE 7:**
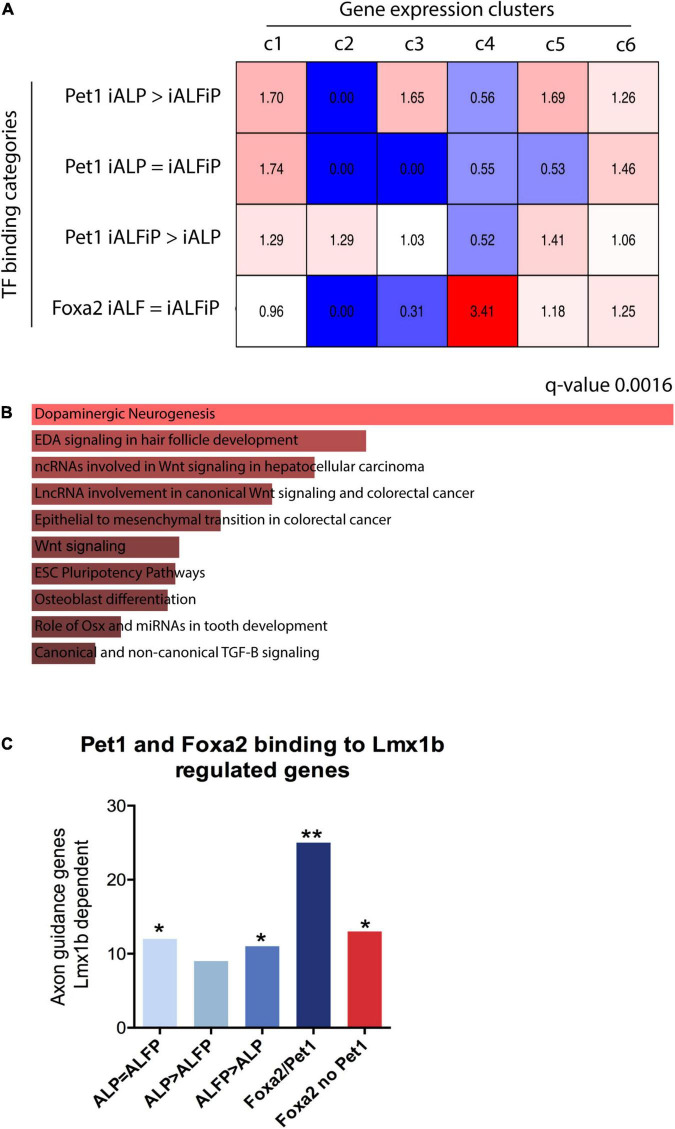
**(A)** Overlap between bulk RNA-seq clusters from Figure 2 48 h and transcription factor binding sites from ChIP-seq experiments. Numbers represent over- and under-representation factors compared with randomly selected regions. **(B)** Gene Ontology analysis for genes bound by Foxa2 in cluster 4 shows enrichment for dopaminergic neurogenesis. **(C)** Overlap between specific binding categories and known serotonergic targets of Lmx1b shows that genes associated to Foxa1/Pet1 co-bound sites are highly enriched for serotonergic functions. *p < 0.5, **p < 0.05.

To assess whether the binding patterns of Foxa2 and Pet1 are associated with the expression patterns unique to iALFiP, we analyzed their gene associations using GREAT (McLean et al., 2010). As shown in Figure 7A, Foxa2’s binding sites are highly associated with genes that are specifically upregulated at 48 h by TF combinations that include Foxa2 (Figure 2D; cluster 4). These genes display upregulation in both iALF and iALFiP and are significantly associated with dopaminergic neurogenesis pathway genes according to Enrichr (Figure 7B). Other categories of binding sites show relatively weaker associations with gene expression categories.

Finally, we directly analyzed the binding patterns of Foxa2 and Pet1 in genes with axonal functions that are known downstream targets of Lmx1b serotonergic terminal selector (Donovan et al., 2019). We selected the top 500 genes associated to each class of binding sites: (1) Pet1 binding ALP = ALFiP; (2) Pet1 binding ALP > ALFiP; (3) Pet1 binding ALP < ALFiP, (4) Pet1/Foxa2 shared sites and (5) Foxa2 binding not co-bound with Pet1. All binding categories are enriched for Lmx1b downstream targets, however the Foxa2/Pet1 bound genes show higher enrichment than considering Pet1 or Foxa2 binding alone (Figure 7C). These results suggest both dependent and independent binding of Pet1 and Foxa2 are important for correct serotonergic differentiation at early differentiation stages.

## Conclusion and Limitations

Transcription factors are potent inducers of gene expression and are thus popularly used to control cell fate for research and clinical applications. This work aimed to understand how TF combinations control specific neuronal fates. To that end, we took advantage of a TF set that contains TFs associated with monoaminergic neuronal fate and proposed to induce serotonergic neuronal fate (Xu et al., 2016). By dissecting the Ascl1 + Lmx1b + Foxa2 + Pet1 (iALFP) combination at the transcriptional output level, combined with how the Pet1 and Foxa2 TFs bind to the genome, we concluded that Pet1 and Foxa2 synergize to induce serotonergic gene expression by binding to some common but mostly distinct sites in the genome. While Foxa2 behaves as a pioneer TF, binds to the same targets in both combinations and increases chromatin accessibility, Pet1 binding is variable. Moreover, Pet1 does not seem to increase chromatin accessibility upon binding. In mouse serotonergic neurons the majority of Pet1 bound regions decrease their accessibility in Pet1 mutants (Zhang et al., 2022). Our data suggests Pet1 could be required in accessibility maintenance rather than acting as a pioneer factor.

Forced TF expression is a standard tool used to investigate TF activity in gain-of-function experiments and laboratory attempts to control cell fate. It is not surprising that Ascl1 induces neuronal fate from pluripotent cells since it has been shown to be sufficient to differentiate stem cells, glia and fibroblast into neurons (Vierbuchen et al., 2010; Raposo et al., 2015; Aydin et al., 2019). With different degrees of success, pro-neuronal TFs such as Ascl1 and Neurog2 were combined with other TFs to canalize differentiation into specific neuronal types (reviewed in Aydin and Mazzoni, 2019). For example, Neurog2 expression alone drives mouse stem cells into a set of possible cortical neuronal identities (Aydin et al., 2019), and pairing Neurog2 with Isl1 and Lhx3 forces most differentiating neurons to become spinal motor neurons (Hester et al., 2011; Mazzoni et al., 2013). While iALFP increases the levels of serotonergic neurons, no combination we tested was able to produce a homogenous culture of 5HT positive neurons. Allowing cultures to mature was enough to canalize the originally dispersed Neurog2-induced neurons into a specific fate (Lu et al., 2019). Similarly, we find better serotonin effector gene expression after 9 days compared to 48 h. Long-term culture might enable iALFP cells to coalesce into a stronger serotonergic fate. Another common limitation of direct programming strategies rests on the TF combination. Here we focused on deconvolving the action of 4 different TFs. However, dozens of TFs are coexpressed in each neuronal type. Further work in basic serotoninergic differentiation mechanisms might produce a new TF set with robust induction capabilities.

We should also consider that induced programming does not reproduce the temporal TF cascade during embryonic differentiation. *In vivo*, TF temporal progression is tightly regulated along the developmental history of a neuron, and this temporal axis might be critical in selecting specific target genes. Indeed, *in vivo*, Ascl1 and Foxa2 are expressed in progenitors, while Lmx1b and Pet1 are expressed and maintained in postmitotic neurons. Thus, Pet1 and Foxa2 are only ephemerally coexpressed in serotonergic neurons *in vivo* while constantly coexpressed during direct programming. Our *in vitro* results show limited Foxa2 and Pet1 direct co-binding, which might reflect *in vivo* gene regulatory networks. Nevertheless, Foxa2 strongly modifies the Pet1 binding landscape during programming, probably through induction of additional downstream TFs. Pet1 controls the expression of different sets of genes during serotoninergic neuron maturation, from axon elongation to axonal branching or neuronal maturation (Wyler et al., 2016; Donovan et al., 2019). Thus, Foxa2 indirect Pet1 relocation could guide Pet1 transitions between stage-specific functions during neuronal maturation. We also want to highlight that despite the low number of Pet1 and Foxa2 co-bound targets, they seem to be biologically relevant as they are highly enriched for genes coding for axonal components that are downstream of the Lmx1b serotonergic terminal selector (which is also known to regulate Pet1 expression itself) (Donovan et al., 2019).

Foxa2 is a well-known pioneer TF, so it makes sense that its binding does not depend on the presence of Pet1. Before the studies presented here, we hypothesized that Pet1 binding would gravitate toward Foxa2 accessible sites. However, our results suggest that Pet1 and Foxa2 synergize to induce serotoninergic fate mostly by binding to different regulatory elements. This implies that establishing general rules that predict the programming abilities of different TF combinations may be challenging. Unlike the clear differences in sequence preference when Isl1 partners with Lhx3 vs. Phox2a (Mazzoni et al., 2013), we did not detect rules that predict Pet1 binding when expressed with Foxa2. As stated above, unknown TFs may co-bind with Pet1 and play a role in producing the transcriptional output generated by Foxa2 + Pet1. Together, this work suggests that each TF combination has its own nuances. Analyzing more examples will produce generalizable rules governing TF binding, leading to the production of specific neuronal subtypes.

## Materials and Methods

### Experimental Procedures

#### Cell Line Generation and Cell Differentiation

Inducible cell lines were generated using a previously described inducible cassette exchange (ICE) method (Iacovino et al., 2011). Resulting transgenic lines contain a single-copy insertion of the transgenes into the HPRT locus that is expression competent. p2Lox-Ascl1 (iAscl1) plasmid was generated by cloning mouse Ascl1 cDNA into p2Lox-V5 plasmid. Likewise, the additional transcription factors were cloned by amplifying open reading frames with p2a or t2a linker peptides as shown in Figure 1. Lmx1b sequence was V5-tagged in iAL and FLAG-tagged at the C-terminal in iALF, iALP, and iALFP, and iALFiP combinations to facilitate immunoprecipitation for ChIP experiments and asses induction efficiency by antibody staining. Pet1 was also V5-tagged for ChIP experiments. Second tetracycline response element (TRE) containing inducible line was generated by inserting TRE-Pet1-HA construct into p2Lox-ALF plasmid which allows two separate TRE elements to control expression of ALF vs Pet1 constructs. HA-tag was added to second TRE Pet1 construct to facilitate ChIP experiments.

Tubb3:T2A-GFPnls ESC knock-in cell line used in sc-RNA-seq experiment was made as described previously (Aydin et al., 2019). The p2Lox-Ascl1 plasmid was nucleofected to Tubb3:T2A-GFPnls ESC line to generate iAscl1 Tubb3:GFP stable line.

The inducible mESCs were grown in 2i (2-inhibitors) based medium Advanced DMEM/F12: Neurobasal (1:1) Medium (Gibco), supplemented with 2.5% mESC-grade fetal bovine serum (vol/vol, Corning), N2 (Gibco), B27 (Gibco), 2 mM L-glutamine (Gibco), 0.1 mM ß-mercaptoethanol (Gibco), 1,000 U ml–1 leukemia inhibitory factor (Millipore), 3 mM CHIR (BioVision) and 1 mM PD0325901 (Sigma) on 0.1% gelatin (Milipore) coated plates at 37 °C, 8% CO2. To generate embryoid bodies (EBs), mESCs were dissociated using TrpLE (Gibco) and plated in AK medium Advanced DMEM/F12: Neurobasal (1:1) Medium, 10% Knockout SR (vol/vol) (Gibco), penicillin–streptomycin (Gibco), 2 mM L-glutamine and 0.1 mM (ß-mercaptoethanol) on untreated plates for 2 days (day –2) at 37 °C, 8% CO2. After 2 days, the expression of the transgenes was induced by adding 3 μg ml–1 doxycycline (Sigma, D9891) to the AK medium. For differentiating mESC (EB) antibody stainings, RNA-seq, sc-RNA-seq, and ATAC-seq experiments, 2–3 × 105 cells were plated in each 100-mm untreated dishes (Corning). For ChIP-seq experiments, the same conditions were used, but the seeded cell number was scaled up to 3–3.5 × 106 cells in 245mm × 245mm square dishes (Corning). For day 9 attached neuron antibody stainings, bulk RNA-seq, ATAC-seq experiments, EBs induced with doxycycline for 2 days (48h + doxycycline) were dissociated with 0.05% Trypsin-EDTA (Gibco) and plated on poly-D-lysine (Sigma, P0899) on coated 4-well plates. The dissociated neurons were grown in neuronal medium with supplements [Neurobasal Medium supplemented with 2% fetal bovine serum, B27, 0.5mM L-glutamine, 0.01mM β-mercaptoethanol, 3 μgml–1 doxycycline, 10 ngml–1 GDNF (PeproTech, 450–10), 10ngml–1 BDNF (PeproTech, 450–02), 10ngml–1 CNTF (PeproTech 450–13), 10 μM Forskolin (Fisher, BP2520–5), and 100 μM IBMX (Tocris, 2845)] at 37Co, 5%CO2. Antimitotic reagents [4 μM 5-fluoro-2’-deoxyuridine (Sigma, F0503) and 4 μM uridine (Sigma, U3003)] were added to eliminate residual proliferating cells.

#### Immunocytochemistry

Embryoid bodies were fixed in 4% paraformaldehyde (vol/vol) in PBS. Fixed EBs were cryoprotected in 30% sucrose and were embedded in OCT (Tissue-Tek) and sectioned for staining. Primary antibody stainings were done by overnight incubation at 4°C, and secondary antibody stainings were incubated for 1 h at room temperature. Day 9 neuronal stainings were done on coverslips coated with poly-D-lysine with the primary and secondary antibody incubation times as described above. Samples were mounted with Fluoroshield with 4,6-diamidino-2phenylindole (DAPI; Sigma) and images were acquired using a SP5 Leica confocal microscope. The following primary and secondary antibodies were used: V5 (Ms): ThermoFisher #R960-25; Tuj1 (MS): Covance #mms-435p; Tuj1 (Rb): Sigma #T2200;5-HT (Rb): Sigma #S5545; 5-HT (Gt): Abcam # Ab66047; TH (Rb): Peel-Freez #P40101-0; TH (Ms): Chemicon #MAB318; TPH1/2 (Sheep): Millipore #AB1541. Alexa 555 anti-mouse: Invitrogen # A31570; Alexa 488 anti-mouse: Invitrogen # A21202; Alexa 633 anti-mouse: Invitrogen # A21052; Alexa 555 anti-rabbit: Invitrogen # A31572; Alexa 555 anti-goat: Invitrogen # A21432; Alexa 488 anti-rabbit: Invitrogen # A21206; Alexa 488 anti-sheep: Invitrogen # A11015.

#### RNA-Seq

Cells were collected in duplicates at 48 h and 9 days after doxycycline induction. We combined new iA RNA-seq with those published (Aydin et al., 2019) to make an n of 5. TRIzol (Invitrogen, 15596026) reagent was used to isolate RNA. Isolated RNA was purified with RNeasy mini kit (Qiagen, 74106). RNA integrity was measured using Agilent High Sensitivity RNA Screentape (Agilent Tech, 5067–5080). 500 ng RNA was spiked (1:100) with ERCC Exfold Spike-in mixes (Thermo Fisher Scientific, 4456739) for accurate comparison across samples. RNA-seq libraries were prepared with Illumina TruSeq LS kit v2 (RS-122–2001; RS-122–2002). KAPA library amplification kit was used for the final quantification of the library before pooling (Roche Lightcycler 480). The libraries were sequenced on an Illumina Next-Seq 500 using V2 and V2.5 chemistry for 50 cycles (single-end) at NYU Genomics Core facility.

#### Single-Cell RNA-Seq

Cells were collected 48 h after doxycycline induction, and washes were done in 1 × PBS with 0.04 mg ml–1 BSA (Thermo Fisher Scientific, AM2616). Clumps were removed by using a 30 μM CellTrics filter (cat. no. 04-004-2326). 25% iA (Tubb3:GFP) and 75% iALF or 25% iA (Tubb3:GFP) and 75% iALFiP were pooled as to separate libraries having 1,000 cells per μl. 10X Genomics Chromium Single Cell 3’ library kit was used to generate a single-cell library for a targeted cell recovery rate of 10,000 cells (Chromium i7 Multiplex Kit, Chromium Single Cell B Chip Kit v3, Chromium Single Cell 3’ GEM, Library and Gel Bead Kit v3). The libraries were sequenced on an Illumina Next-Seq 500 High Output using V2.5 chemistry with 26 × 98 bp – 150 cycles run confirmation at NYU Genomics Core facility.

#### ChIP-Seq

Cells were collected and fixed with 1 mM DSG (ProtoChem) followed by 1% FA (vol/vol) each for 15 min at room temperature. Pellets containing 25–30 × 10^6^ cells were aliquoted and flash-frozen at –80°C. Cells were lysed in lysis buffer containing 50 mM HEPES-KOH (pH 7.5), 140 mM NaCl, 1 mM EDTA, 10% glycerol (vol/vol), 0.5% Igepal (vol/vol), 0.25% Triton X-100 (vol/vol) with 1 × protease inhibitors (Roche, 11697498001) at 4°C for 10 min. Cells were resuspended in 50 mM HEPES-KOH (pH 7.5), 140 mM NaCl, 1 mM EDTA, 10% glycerol (vol/vol), 0.5% Igepal (vol/vol), 0.25% Triton X-100 (vol/vol), and incubated 10 min at 4°C. Nuclear extracts were resuspended in cold sonication buffer [50 mM HEPES (pH 7.5), 140 mM NaCl, 1 mM EDTA, 1 mM EGTA, 1% Triton X-100, 0.1% sodium deoxycholate (wt/vol), 0.1% SDS (wt/vol)]. Sonication was performed with Bioruptor Pico sonicator device (Diagenode) with 30 sec ON/30 sec OFF, 18 cycles, with Bioruptor sonication beads (0.45 mg beads per 1 ml sample). Immunoprecipitation was done overnight at 4°C on a rotator with Dynabeads protein-G (Thermo Fisher Scientific) conjugated antibodies. 5 μg of the following antibodies were used for immunoprecipitation: anti-Ascl1 (Abcam, ab74065); anti-HA (Abcam, ab9110); anti-V5 (Abcam, ab15828). Subsequent washes were done in 1X sonication buffer (cold) first, sonication buffer with 500 nM NaCl (cold), LiCl wash buffer [20 mM Tris−HCl (pH 8.0)] (cold), 1 mM EDTA, 250 mM LiCl, 0.5% NP-40, 0.5% sodium deoxycholate (cold), and TE buffer [10 mM Tris, 1 mM EDTA, (pH 8)] (cold). Samples were eluted in elution buffer [50 mM Tris−HCl (pH 8.0), 10 mM EDTA (pH 8.0), 1% SDS] by incubating for 45 min at 65°C. Eluted sample and input (sonicated only) were incubated overnight at 65°C to reverse the crosslink. RNA was digested by the addition of 0.2 mg ml^–^1 RNase A (Sigma) and incubating for 2 h at 37°C. Protein digestion was performed by adding 0.2 mg ml^–^1 Proteinase K (Invitrogen) for 30 min at 55°C. DNA extraction was done with Phenol:chloroform:isoamyl alcohol (25:24:1; vol/vol) (Invitrogen) followed by ethanol precipitation. 1/3 of ChIP DNA (1:100 dilution of input DNA) was used to prepare lllumina DNA sequencing libraries. Bioo Scientific multiplexed adapters were ligated after end repair and A-tailing, and unligated adapters were removed with Agencourt AmpureXP beads (Beckman Coulter) purification. Adapter-ligated DNA was amplified by PCR using TruSeq primers (Sigma). DNA libraries between 300 and 500bp in size were purified from agarose gel using a Qiagen minElute column, and the final quantification of the library before pooling was done using a KAPA library amplification kit (Roche Lightcycler 480). The libraries were sequenced on an Illumina Next-Seq 500 using V2 chemistry for 50 cycles (single-end) at NYU Genomics Core facility. The experiments were done in duplicate.

#### ATAC-Seq

The 50,000 cells were harvested and washed twice in cold 1X PBS. Cells were resuspended in 10 mM Tris (pH 7.4), 10 mM NaCl, 3 mM MgCl2, and 0.1% NP-40, and centrifuged immediately at 4°C for 10 min. Day 9 attached neuron samples were lyzed in 0.01% NP-40 instead. The pellet was resuspended in 25 μl of 2 × TD buffer, 2.5 μl TDE1 (Nextera DNA sample preparation kit, FC-121– 1030) followed by incubation for 30 min at 37°C. The reaction was cleaned up with Min-elute PCR purification kit (Qiagen, 28004). The optimal number of PCR cycles were determined to be the one-third of the maximum fluorescence measured by quantitative PCR reaction with 1 × SYBR Green (Invitrogen), custom-designed primers (Buenrostro et al., 2013) and 2 × NEB MasterMix (New England Labs, M0541). The library was cleaned up with Min-elute PCR kit and quantified using Qubit (Life Technologies, Q32854). The fragment length distribution of the library was determined using an Agilent High Sensitivity DNA D1000 Screentape (5067–5585) system, and the final quantification of the library before pooling was done using a KAPA library amplification kit (Roche Lightcycler 480). The libraries were sequenced on an Illumina Next-Seq 500 using V2 chemistry for 150 cycles (paired-end 75 bp) at NYU Genomics Core facility. The experiments were done in duplicate.

### Quantification and Statistical Analysis

#### RNA-Seq Data Analysis

All RNA-seq fastq files were aligned to the mouse genome (version mm10) using STAR (Dobin et al., 2013) version 2.7.7a with options: −−outFilterMultimapNmax 10 − −alignSJoverhangMin 8 −−alignSJDBoverhang Min 1 −−out FilterMismatchNmax 999 −−outFilterMismatchNoverReadLmax 0.2 −−align IntronMin 20 −−alignIntronMax 1000000 −− alignMatesGapMax 1000000. Read assignment to genes was performed by the Rsubread (Liao et al., 2019) featureCounts (v2.0.2) command using the GENCODE M20 annotation. DESeq2 (Love et al., 2014) was used to define differentially expressed genes using a *q*-Value cutoff of less than 0.05. K-means clustering was performed using the kmeans package in R. Values of K between 4 and 10 were tested, with 6 offering the best qualitative balance between cluster size and interpretability. Enrichr (Xie et al., 2021) was used to perform gene enrichment analysis. Heatmaps were generated using the ComplexHeatmap (Gu et al., 2016) package in R.

#### Single-Cell RNA-Seq Data Analysis

Fastq files were generated by using CellRanger (v.2.1.0) from 10x Genomics with default settings^1^. A custom reference genome was generated using the CellRanger mkref function by passing the modified FastA and GTF files as described (Aydin et al., 2019) to distinguish the pooled cell lines by adding exogenous sequences to the mm10 reference genome. CellRanger count function was used to generate single cell feature counts for the library. CellRanger merge function was used to merge datasets. Downstream analyses and graph visualizations were performed in Seurat R package (Butler et al., 2018) (v3). Briefly, we removed the cells that have unique gene counts greater than 6,800 (potential doublets) and less than 200. After removing the unwanted cells, we normalized the data by a global-scaling normalization method (logNormalize) with the default scale factor (10,000). Linear dimensional reduction was performed by PCA, and the clustering was performed by using the statistically significant principal components (identified using the jackStraw method and by the standard deviation of principle components). Seurat objects were integrated by FindIntegrationAnchors and IntegrateData functions as described in this tutorial^2^. The results were visualized using UMAP plots.

#### ChIP-Seq Data Analysis

All ChIP-seq fastq files were aligned to the mouse genome (version mm10) using Bowtie (Langmead et al., 2009), with only uniquely mapped reads used for analysis. MultiGPS (Mahony et al., 2014) (version 0.75) was used to define transcription factor DNA binding events, with a cutoff of *q*-Value < 0.01 (using binomial tests and Benjamini-Hochberg multiple hypothesis correction) for designating statistically significant events. Differential binding analysis between different conditions was also performed with MultiGPS, which uses EdgeR (Robinson et al., 2010) internally. Heatmaps were generated using the Deeptools package (Ramirez et al., 2016). Motifs were identified using MEME-ChIP (version 5.3.3) (Machanick and Bailey, 2011) using default parameters.

#### Discriminative Motif Analysis

SeqUnwinder (version 0.1.5) (Kakumanu et al., 2017) was used to find motifs that discriminate between Pet1 binding site categories, using parameters: −−threads 4 −−win 200 −−mink 4 −−maxk 5 −−r 10 −−x 3 −−a 400 −−hillsthresh 0.1 −−memesearchwin 16, and using MEME version 5.3.3 internally.

#### ATAC-Seq Data Analysis

All ATAC-seq fastq files were aligned to the mouse genome (version mm10) using Bowtie (Langmead et al., 2009), with only uniquely mapped reads used for analysis. Heatmaps were plotted using Deeptools (Ramirez et al., 2016).

#### Associations Between Differentially Expressed Genes and Differentially Bound Transcription Factor Binding Sites

The GREAT (McLean et al., 2010) command-line tools were used to define regulatory domains and to assess the associations between ChIP-seq binding locations and differentially expressed genes. The GREAT regulatory domains were defined using the GREAT “basal plus extension” model using settings: basalUpstream = 5000, basalDownstream = 1000, maxExtension = 100000. Overrepresentation was calculated compared to the average & standard deviation of ten sets of randomly selected locations as described previously (Aydin et al., 2019).

## Data Availability Statement

The datasets presented in this study can be found in online repositories. The names of the repository/repositories and accession number(s) can be found below: https://www.ncbi.nlm.nih.gov/geo/query/acc.cgi?acc=GSE199315.

## Author Contributions

BA performed most of the experiments. MS and BA analyzed the data. MM-E and BA made and validated the cell lines with the help of LT and NK. NF, SM, and EM formulated the project and supervised the research. BA, MS, NF, SM, and EM wrote the manuscript. All authors contributed to the article and approved the submitted version.

## Conflict of Interest

The authors declare that the research was conducted in the absence of any commercial or financial relationships that could be construed as a potential conflict of interest.

## Publisher’s Note

All claims expressed in this article are solely those of the authors and do not necessarily represent those of their affiliated organizations, or those of the publisher, the editors and the reviewers. Any product that may be evaluated in this article, or claim that may be made by its manufacturer, is not guaranteed or endorsed by the publisher.

## Footnotes

1
https://support.10xgenomics.com/single-cell-gene-expression/software/pipelines/latest/what-is-cell-ranger

2
https://satijalab.org/seurat/v3.0/immune_alignment.html

## Supplementary Material

Supplementary Figure 1 **(A)** Western bot of all inducible lines 48 h after induction revealed with anti-V5 antibody for the last TF in each combination. **(B)** AFPKMs of selected markers. **(C)** Volcano plots of gene expression under various exogenous transcription factor constructs at 48 h and 9 days post-induction. All plots are differential expression compared to embryonic bodies. Dashed lines show the *p*-Value cutoff (<0.05) and log fold change cutoff (≥ 1.0). Red points pass both cutoffs, blue points pass the *p*-Value cutoff, green points pass the log fold change cutoff, and gray points pass neither cutoff. Some specific diagnostic genes are labeled and are plotted as a triangle. Plot was made with the R EnhancedVolcano package. **(D,E)** Bulk RNA-seq heatmaps under different exogenous transcription factor constructs at 48 h **(D)** and 9 days **(E)** post-induction. In contrast to the filtered sets of genes shown in Figure 2, these plots display a K-means clustering (using the R kmeans function) of all genes that are significantly differentially expressed in one or more conditions. **(F)** Venn diagrams comparing the differential expression of the lines. Red: Genes upregulated, Blue: Genes downregulated.

Supplementary Figure 2 **(A)** Motif enrichment at Pet1 binding sites. **(B)** Foxa2 and Pet1 binding distribution.

Supplementary Figure 3 Lmx1b ChiP-seq analysis.

Supplementary Table 1 Raw scoring data for Figure 1.

Supplementary Table 2 Genes names for clusters in Figure 2.

Supplementary Table 3 GO terms associated with clusters in Figure 2 48 hs.

Supplementary Table 4 GO terms associated with clusters in Figure 2 Day 9.
