# Supplementary material for: Foxa2 and Pet1 Direct and Indirect Synergy Drive Serotonergic Neuronal Differentiation
Source: Front Neurosci. 2022 Jun 20;16:903881. doi: 10.3389/fnins.2022.903881 (PMC9254625; doi:10.3389/fnins.2022.903881)
Supplement: Supplementary Figure 1 — (A) Western bot of all inducible lines 48 h after induction revealed with anti-V5 antibody for the last TF in each combination. (B) AFPKMs of selected markers. (C) Volcano plots of gene expression under various exogenous transcription factor constructs at 48 h and 9 days post-induction. All plots are differential expression compared to embryonic bodies. Dashed lines show the p-Value cutoff (<0.05) and log fold change cutoff (≥ 1.0). Red points pass both cutoffs, blue points pass the p-Value cutoff, green points pass the log fold change cutoff, and gray points pass neither cutoff. Some specific diagnostic genes are labeled and are plotted as a triangle. Plot was made with the R EnhancedVolcano package. (D,E) Bulk RNA-seq heatmaps under different exogenous transcription factor constructs at 48 h (D) and 9 days (E) post-induction. In contrast to the filtered sets of genes shown in Figure 2, these plots display a K-means clustering (using the R kmeans function) of all genes that are significantly differentially expressed in one or more conditions. (F) Venn diagrams comparing the differential expression of the lines. Red: Genes upregulated, Blue: Genes downregulated. [file Image_1.pdf]

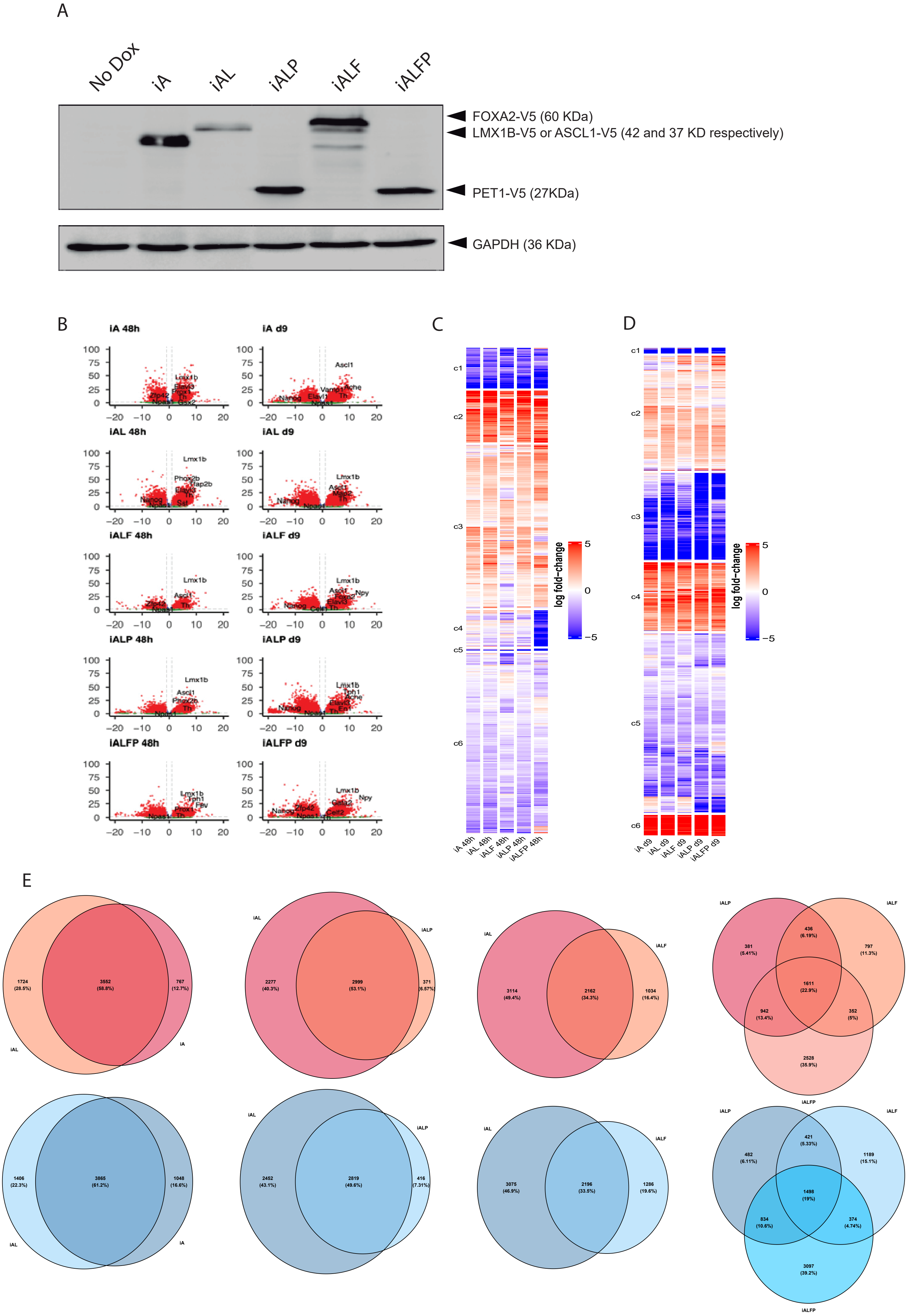

A
No Dox
iA
iAL
iALP
iALF
iALFP
FOXA2-V5 (60 KDa)
LMX1B-V5 or ASCL1-V5 (42 and 37 KD respectively)
PET1-V5 (27KDa)
GAPDH (36 KDa)
B
iA 48h
iA d9
iAL 48h
iAL d9
iALF 48h
iALF d9
iALP 48h
iALP d9
iALFP 48h
iALFP d9
C
D
log fold-change
iA 48h
iAL 48h
iALF 48h
iALP 48h
iALFP 48h
iA d9
iAL d9
iALF d9
iALP d9
iALFP d9
E

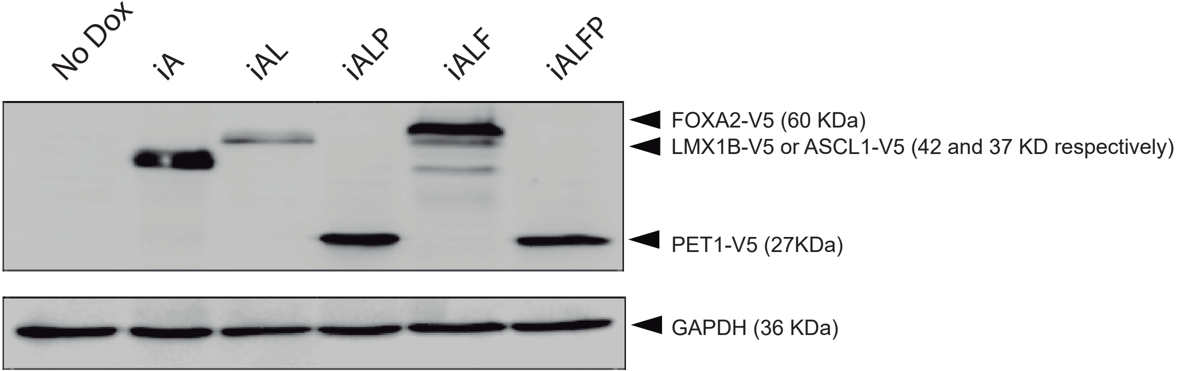
No Dox
iA
iAL
iALP
iALF
iALFP
FOXA2-V5 (60 KDa)
LMX1B-V5 or ASCL1-V5 (42 and 37 KD respectively)
PET1-V5 (27KDa)
GAPDH (36 KDa)
