# Supplementary figures and images for: Foxa2 and Pet1 Direct and Indirect Synergy Drive Serotonergic Neuronal Differentiation
Source: Front Neurosci. 2022 Jun 20;16:903881. doi: 10.3389/fnins.2022.903881 (PMC9254625; doi:10.3389/fnins.2022.903881)

A

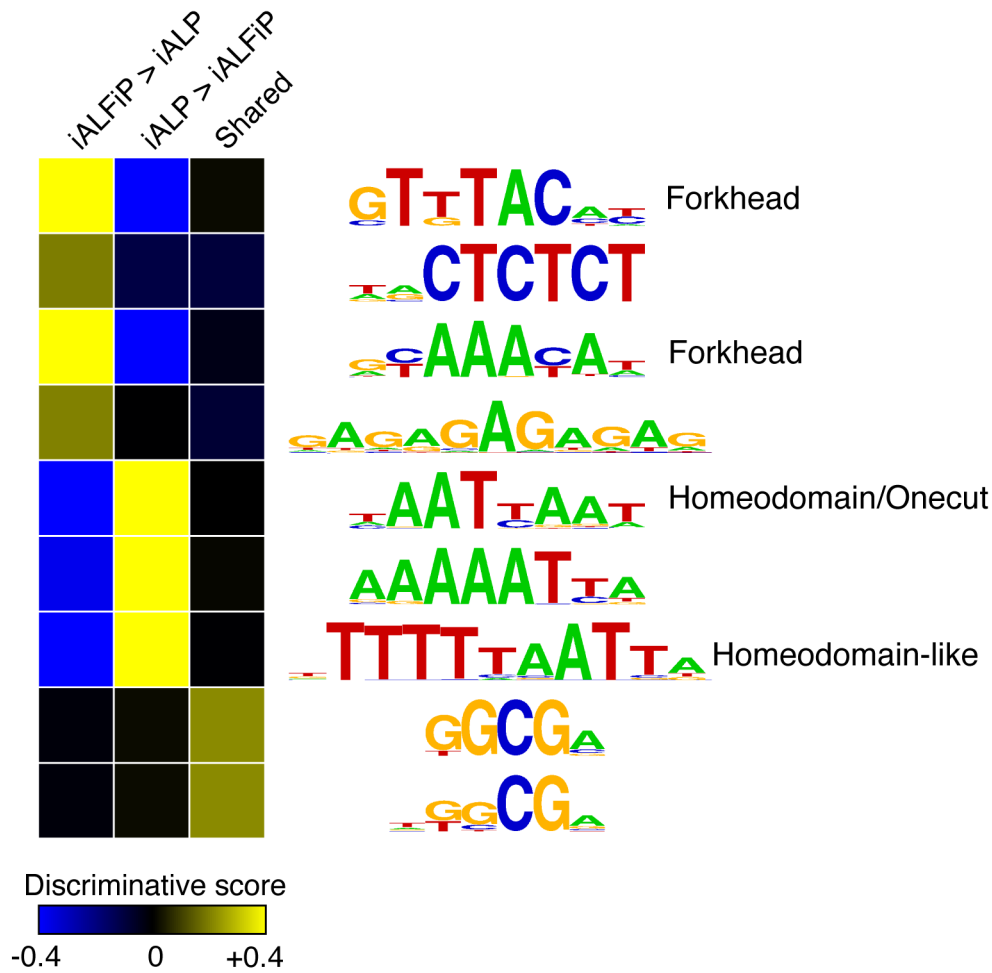

B

Foxa2

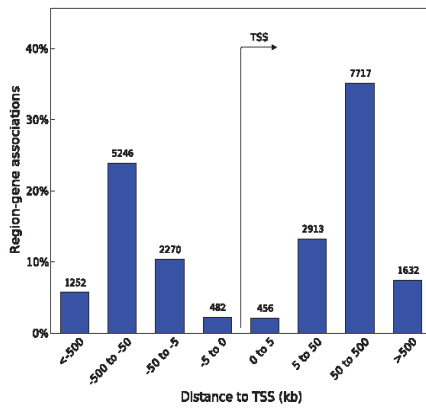

Pet1

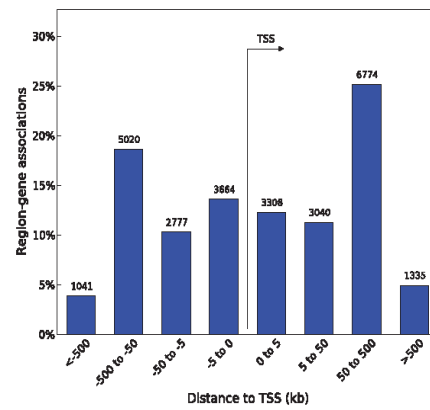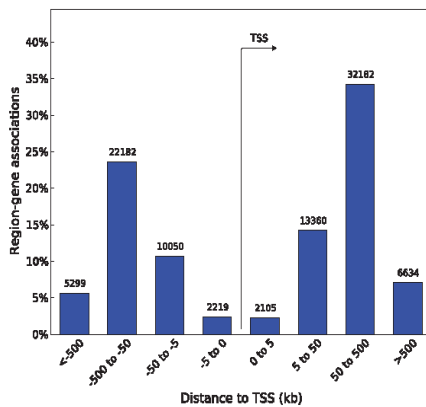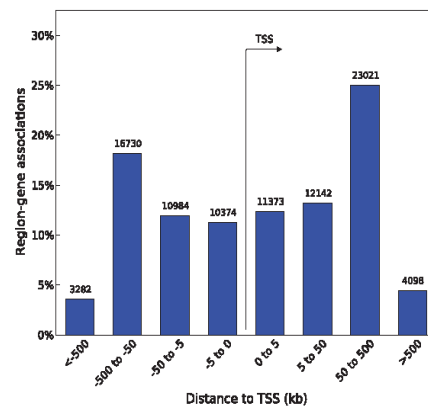

Supplement: Supplementary Figure 2 — (A) Motif enrichment at Pet1 binding sites. (B) Foxa2 and Pet1 binding distribution. [file Image_2.pdf]

A) Lmx1b  
(iAL 48hrs)

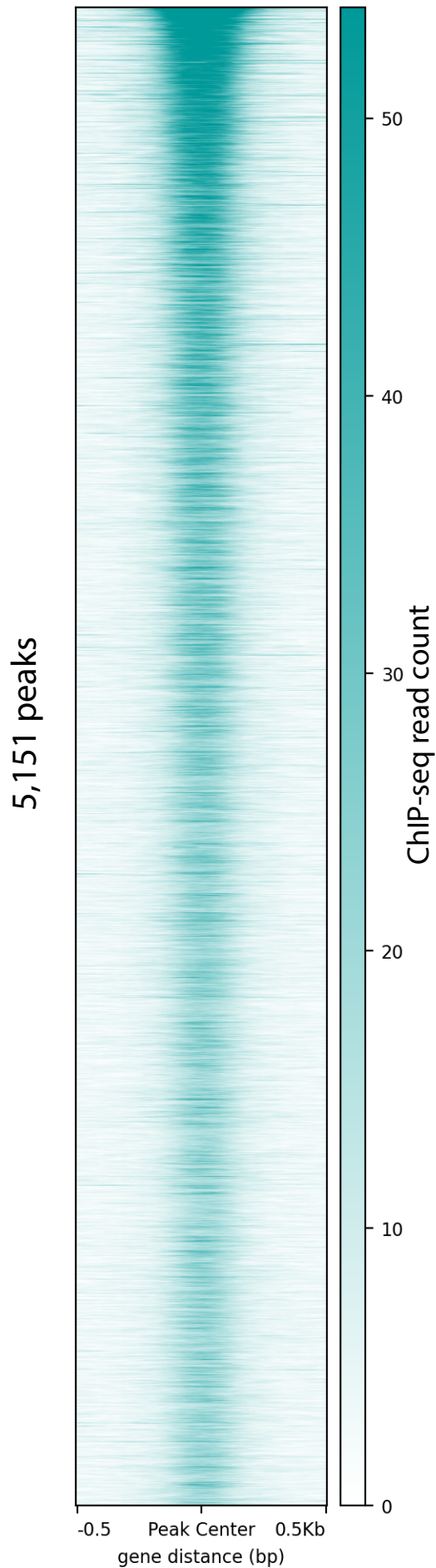

B) MEME-ChIP motifs  
in top 1,000 peaks

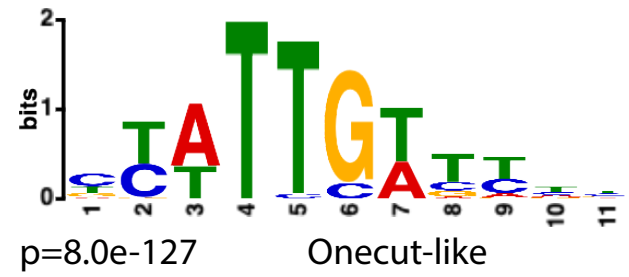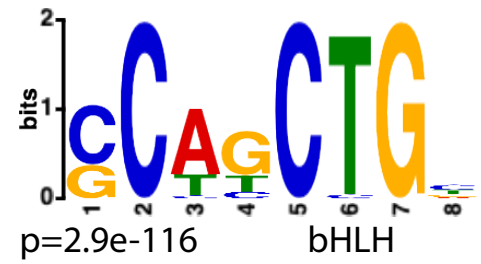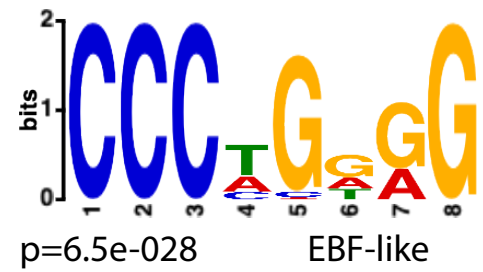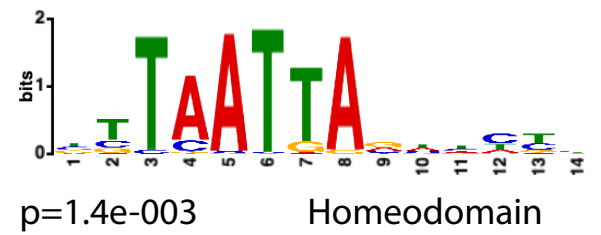

Supplement: Supplementary Figure 3 — Lmx1b ChiP-seq analysis. [file Image_3.pdf]
